# Supplementary material for: Protein engineering expands the effector recognition profile of a rice NLR immune receptor
Source: eLife. 2019 Sep 19;8:e47713. doi: 10.7554/eLife.47713 (PMC6768660; doi:10.7554/eLife.47713)
Supplement: Supplementary file 1. — Underlined values are those presented in the respective figures. [file elife-47713-supp1.docx]

**Supplementary Table 1: p-values for all pairwise comparisons of the ion leakage data in *N. benthamiana*.** Underlined values are those presented in the respective Figures.

**Pikp vs. Pikp^NK-KE^:**

| **p values from statistical analysis and Tukey’s HSD: Ion leakage assay** | | | | | | | | |
| --- | --- | --- | --- | --- | --- | --- | --- | --- |
|  | **Pikp/ AVR-PikD** | **Pikp/ AVR-PikE** | **Pikp/ AVR-PikA** | **Pikp/ AVR-PikC** | **Pikp^NK-KE^/ AVR-PikD** | **Pikp^NK-KE^/ AVR-PikE** | **Pikp^NK-KE^/ AVR-PikA** | **Pikp^NK-KE^/ AVR-PikC** |
| **Pikp/ AVR-PikD** |  | **7.26E-11** | **7.72E-11** | **5.19E-11** | **5.41E-04** | **7.19E-04** | **1.17E-3** | **6.48E-11** |
| **Pikp/  AVR-PikE** | **7.26E-11** |  | **1** | **1** | **3.61E-12** | **2.76E-3** | **1.72E-3** | **1** |
| **Pikp/  AVR-PikA** | **7.72E-11** | **1** |  | **1** | **3.61E-12** | **2.93E-3** | **1.82E-3** | **1** |
| **Pikp/  AVR-PikC** | **5.19E-11** | **1** | **1** |  | **3.61E-12** | **2.01E-3** | **1.25E-3** | **1** |
| **Pikp^NK-KE^/ AVR-PikD** | **5.41E-04** | **3.61E-12** | **3.61E-12** | **3.61E-12** |  | **1.53E-11** | **2.33E-11** | **3.61E-12** |
| **Pikp^NK-KE^/ AVR-PikE** | **7.19E-04** | **2.76E-3** | **2.93E-3** | **2.01E-3** | **1.53E-11** |  | **1** | **2.49E-3** |
| **Pikp^NK-KE^/ AVR-PikA** | **1.17E-3** | **1.72E-3** | **1.82E-3** | **1.25E-3** | **2.33E-11** | **1** |  | **1.55E-3** |
| **Pikp^NK-KE^/ AVR-PikC** | **6.48E-11** | **1** | **1** | **1** | **3.61E-12** | **2.49E-3** | **1.55E-3** |  |

**Pikm vs. Pikp^NK-KE^:**

| **p values from statistical analysis and Tukey’s HSD: Ion leakage assay** | | | | | | | | |
| --- | --- | --- | --- | --- | --- | --- | --- | --- |
|  | **Pikm/  AVR-PikD** | **Pikm/  AVR-PikE** | **Pikm/  AVR-PikA** | **Pikm/  AVR-PikC** | **Pikp^NK-KE^/ AVR-PikD** | **Pikp^NK-KE^/ AVR-PikE** | **Pikp^NK-KE^/ AVR-PikA** | **Pikp^NK-KE^/ AVR-PikC** |
| **Pikm/  AVR-PikD** |  | **2.75E-05** | **1.55E-06** | **2.91E-11** | **1.61E-3** | **2.35E-04** | **3.89E-04** | **2.25E-11** |
| **Pikm/  AVR-PikE** | **2.75E-05** |  | **1** | **0.02** | **5.05E-12** | **1** | **1** | **1.60E-2** |
| **Pikm/  AVR-PikA** | **1.55E-06** | **1** |  | **0.15** | **3.76E-12** | **0.96** | **0.93** | **0.12** |
| **Pikm/  AVR-PikC** | **2.91E-11** | **0.02** | **0.15** |  | **3.61E-12** | **3.26E-3** | **2.03E-3** | **1** |
| **Pikp^NK-KE^/ AVR-PikD** | **1.61E-3** | **5.05E-12** | **3.76E-12** | **3.61E-12** |  | **1.53E-11** | **2.33E-11** | **3.61E-12** |
| **Pikp^NK-KE^/ AVR-PikE** | **2.35E-04** | **1** | **0.96** | **3.26E-3** | **1.53E-11** |  | **1** | **2.49E-3** |
| **Pikp^NK-KE^/ AVR-PikA** | **3.89E-04** | **1** | **0.93** | **2.03E-3** | **2.33E-11** | **1** |  | **1.55E-3** |
| **Pikp^NK-KE^/ AVR-PikC** | **2.25E-11** | **1.60E-2** | **0.12** | **1** | **3.61E-12** | **2.49E-3** | **1.55E-3** |  |
