# Supplementary material for: Protein engineering expands the effector recognition profile of a rice NLR immune receptor
Source: eLife. 2019 Sep 19;8:e47713. doi: 10.7554/eLife.47713 (PMC6768660; doi:10.7554/eLife.47713)
Supplement: Supplementary file 2. — Underlined values are those presented in the respective figures. [file elife-47713-supp2.docx]

**Supplementary Table 2: p-values for all pairwise comparisons of the SPR data including Pikp and Pikp^NK-KE^.** Underlined values are those presented in the respective Figures.

**Pikp vs. Pikp^NK-KE^:**

| **p values from statistical analysis and Tukey’s HSD: SPR** | | | | | | | | |
| --- | --- | --- | --- | --- | --- | --- | --- | --- |
|  | **Pikp/  AVR-PikD** | **Pikp/  AVR-PikE** | **Pikp/ AVR-PikA** | **Pikp/  AVR-PikC** | **Pikp^NK-KE^/ AVR-PikD** | **Pikp^NK-KE^/ AVR-PikE** | **Pikp^NK-KE^/ AVR-PikA** | **Pikp^NK-KE^/ AVR-PikC** |
| **Pikp/  AVR-PikD** |  | **2.46E-04** | **1.10E-06** | **7.86E-08** | **1** | **0.11** | **3.07E-02** | **1.58E-03** |
| **Pikp/  AVR-PikE** | **2.46E-04** |  | **2.48E-02** | **3.56E-04** | **1.09E-04** | **5.20E-02** | **0.18** | **0.94** |
| **Pikp/  AVR-PikA** | **1.10E-06** | **2.48E-02** |  | **0.30** | **6.03E-07** | **6.66E-05** | **2.03E-04** | **3.35E-03** |
| **Pikp/  AVR-PikC** | **7.86E-08** | **3.56E-04** | **0.30** |  | **4.72E-08** | **2.40E-06** | **6.03E-06** | **6.23E-05** |
| **Pikp^NK-KE^/ AVR-PikD** | **1** | **1.09E-04** | **6.03E-07** | **4.72E-08** |  | **4.43E-02** | **1.21E-02** | **6.55E-04** |
| **Pikp^NK-KE^/ AVR-PikE** | **0.11** | **5.20E-02** | **6.66E-05** | **2.40E-06** | **4.43E-02** |  | **1** | **0.32** |
| **Pikp^NK-KE^/ AVR-PikA** | **3.07E-02** | **0.18** | **2.03E-04** | **6.03E-06** | **1.21E-02** | **1** |  | **0.71** |
| **Pikp^NK-KE^/ AVR-PikC** | **1.58E-03** | **0.94** | **3.35E-03** | **6.23E-05** | **6.55E-04** | **0.32** | **0.71** |  |

**Pikm vs. Pikp^NK-KE^:**

| **p values from statistical analysis and Tukey’s HSD: SPR** | | | | | | | | |
| --- | --- | --- | --- | --- | --- | --- | --- | --- |
|  | **Pikm/ AVR-PikD** | **Pikm/  AVR-PikE** | **Pikm/  AVR-PikA** | **Pikm/  AVR-PikC** | **Pikp^NK-KE^/ AVR-PikD** | **Pikp^NK-KE^/ AVR-PikE** | **Pikp^NK-KE^/ AVR-PikA** | **Pikp^NK-KE^/ AVR-PikC** |
| **Pikm/  AVR-PikD** |  | **8.28E-04** | **1.61E-05** | **9.92E-07** | **1** | **2.66E-02** | **8.92E-03** | **9.02E-04** |
| **Pikm/  AVR-PikE** | **8.28E-04** |  | **4.17E-02** | **3.34E-04** | **7.31E-04** | **0.13** | **0.37** | **1** |
| **Pikm/  AVR-PikA** | **1.61E-05** | **4.17E-02** |  | **5.56E-02** | **1.16E-05** | **4.75E-04** | **1.18E-03** | **1.21E-02** |
| **Pikm/  AVR-PikC** | **9.92E-07** | **3.34E-04** | **5.56E-02** |  | **6.54E-07** | **1.02E-05** | **1.94E-05** | **1.01E-04** |
| **Pikp^NK-KE^/ AVR-PikD** | **1** | **7.31E-04** | **1.16E-05** | **6.54E-07** |  | **2.16E-02** | **6.31E-03** | **5.02E-04** |
| **Pikp^NK-KE^/ AVR-PikE** | **2.66E-02** | **0.13** | **4.75E-04** | **1.02E-05** | **2.16E-02** |  | **0.98** | **0.18** |
| **Pikp^NK-KE^/ AVR-PikA** | **8.92E-03** | **0.37** | **1.18E-03** | **1.94E-05** | **6.31E-03** | **0.98** |  | **0.52** |
| **Pikp^NK-KE^/ AVR-PikC** | **9.02E-04** | **1** | **1.21E-02** | **1.01E-04** | **5.02E-04** | **0.18** | **0.52** |  |
